# Language Difficulties in School-Age Children With Developmental Dyslexia

**DOI:** 10.1177/00222194211006207

**Published:** 2021-04-23

**Authors:** Kaitlyn M. Price, Karen G. Wigg, Virginia L. Misener, Antoine Clarke, Natalie Yeung, Kirsten Blokland, Margaret Wilkinson, Elizabeth N. Kerr, Sharon L. Guger, Maureen W. Lovett, Cathy L. Barr

**Affiliations:** 1 University Health Network, Toronto, Ontario, Canada; 2 The Hospital for Sick Children, Toronto, Ontario, Canada; 3 University of Toronto, Ontario, Canada

**Keywords:** dyslexia, reading disabilities, early language delay, expressive/receptive language difficulties

## Abstract

Developmental dyslexia (DD) is a common reading disability, affecting 5% to 11% of children in North America. Children classified as having DD often have a history of early language delay (ELD) or language impairments. Nevertheless, studies have reported conflicting results as to the association between DD-ELD and the extent of current language difficulties in children with DD. To examine these relationships, we queried the parents of school-age children with reading difficulties on their child’s early and current language ability. Siblings were also examined. Children were directly assessed using quantitative tests of language and reading skills. To compare this study with the literature, we divided the sample (*N* = 674) into three groups: DD, intermediate readers (IR), and skilled readers (SR). We found a significant association between DD and ELD, with parents of children in the DD/IR groups reporting their children put words together later than the SR group. We also found a significant association between DD and language difficulties, with children with low reading skills having low expressive/receptive language abilities. Finally, we identified early language predicted current language, which predicted reading skills. These data contribute to research indicating that children with DD experience language difficulties, suggesting early recognition may help identify reading problems.

Developmental dyslexia (DD) is a developmental neurocognitive disorder affecting 5% to 11% of children in North America (Katusic et al., 2001; Shaywitz & Shaywitz, 2005; Snowling & Melby-Lervag, 2016). It is defined by “difficulties with accurate/fluent word recognition and by poor spelling and decoding abilities” (Lyon, 2003, p. 2). DD is characterized by difficulties in phonological decoding and phoneme awareness (Lyon, 2003; Perfetti, 1985). Phonological decoding and phoneme awareness precede single-word reading and are the basic foundational skills for the development of reading comprehension. As such, DD may lead to secondary problems, including difficulties with reading fluency and comprehension (Lyon, 2003). In the context of this study, we will be discussing children specifically measured for DD (decoding/word identification).

Children classified as having DD often have a higher prevalence of language difficulties, including early language delay (ELD) and language impairments (LIs). Within the literature, ELD is defined by a failure to combine words by 1.5 to 3 years old, or by vocabulary below the 10th percentile of norms at age 2 to 4 years (Poll & Miller, 2013; Reilly et al., 2010; Rice et al., 2008). Language impairment is defined as “difficulties in the acquisition and use of language across modalities (i.e., spoken, written, sign language, or other)” (American Psychiatric Association [APA], 2013, p. 42). These difficulties in language are due to problems with comprehension or production, in the absence of hearing loss or other developmental delay (APA, 2013; Pennington & Bishop, 2009). It includes expressive and receptive language difficulties. Expressive language difficulties are defined by problems producing language to convey thoughts, information, and feelings. Receptive language difficulties are defined by problems understanding instructions or conversation (McLaughlin, 2011).

Although defined categorically, the characteristics described for DD, ELD, and LI are found on a continuum, with children often struggling in more than one domain. For three decades, studies have examined the relationship between DD and ELD and DD and LI (reviewed by McArthur et al., 2000; Pennington & Bishop, 2009; Rescorla, 2011); however, evidence for the association between DD and ELD differs across studies, and questions remain as to the extent of current language difficulties in children classified as having DD.

Beginning with the association between DD and ELD, the consensus is that ELD puts a child at greater risk of developing reading difficulties compared to children with typically developing language, despite the fact this has not been universally found (Rescorla, 2011). A significant association was demonstrated in a sample of 16 children followed from age 2.5 to 7 years old (Scarborough & Dobrich, 1990) and was supported by larger studies. For example, a study of 174 children by Preston and colleagues (2010) and a study of 200 children by Lyytinen and colleagues (2005) examined 4 to 12 year olds with a family history of reading/language difficulties; both reports found an association between DD and ELD (Lyytinen et al., 2005; Preston et al., 2010). However, the DD-ELD association has been challenged by studies that have not found this significant link. The nonsignificant findings were from samples of children ages 4 to 17 years old with a history of ELD and ranged in size from fewer than 60 (Duff et al., 2015; Paul et al., 1997; Rescorla, 2005, 2009) to greater than 1,000 participants (Dale et al., 2014; Poll & Miller, 2013). It should be noted that although Duff et al. (2015) did not find significant differences between children with a history of ELD, measured by vocabulary, at 18 months and children with typical development, they did report a significant difference between children with LI at age 4 and children with typical development on accuracy of reading at age 7.

The differing results of DD-ELD association studies may be attributable to differing degrees of language deficits or reading levels sampled across different studies. It has been found that a large proportion of children with ELD will “catch up” to national normative values not only on reading measures but also on language measures by 5 to 7 years of age (Rescorla, 2011). Therefore, although ELD is considered to be a risk factor for later language problems, some of these children’s language deficits will resolve over time. These differences in language levels could affect reading skills. Other children may score within national norms for language and reading but score significantly lower than their typically developing peers (Rescorla, 2011). Furthermore, studies often measure reading at different ages, which can affect association estimates. For example, Rescorla (2002) examined 59 children ages 6 to 9 years and found that at ages 6 and 7, children with ELD and children with typical development did not differ in reading skills (word reading); however, when they followed the same group to ages 8 and 9, the late talkers performed more poorly than controls. Based on this, Rescorla (2002) concluded that impairment in reading skills became more apparent as reading demands increased in school.

Next, although a great deal of research supports a relationship between DD and LI (Catts et al., 2005; Pennington & Bishop, 2009; Snowling & Melby-Lervag, 2016; Tomblin et al., 1997), the extent of language difficulties in children with DD—and overlap reported—varies between studies. The DD-LI association has been reported for both expressive and receptive language difficulties (Torppa et al., 2010), with expressive vocabulary knowledge predicting word identification skills and receptive vocabulary knowledge predicting reading-related skills, defined as phonological processes and sound symbol identification (reviewed by Wise et al., 2007). Support for the association between DD and LI initially came from large studies, consisting of up to 500 participants, which typically recruited children ages 4 to 6 years old with language difficulties and followed them longitudinally to assess later reading development. They reported that the LI group performed more poorly on reading measures than the typically developing language group (Aram & Nation, 1980; Bishop & Adams, 1990; Catts et al., 2002, 2005; Goulandris et al., 2000; Snowling et al., 2000, 2016; Stark et al., 1984). Some of this research also included children with a family history of reading difficulties or a DD group (Catts et al., 2005; Goulandris et al., 2000; Snowling et al., 2016; for review of family history studies, see Adlof & Hogan, 2018; Snowling & Melby-Lervag, 2016). Further support for the DD-LI association came from studies that investigated language in children ascertained for reading difficulties (reviewed by McArthur et al., 2000) or reading population-based twin studies (DeThorne et al., 2006).

Although DD has been significantly associated with lower language measures and LI with lower reading measures, differing results have been reported. For example, Alt et al. (2017) and Catts et al. (2005) found children with DD scored significantly different from the typically developing group on language measures but still within national norms (Alt et al., 2017; Catts et al., 2005; this was an inclusion criterion in the Alt study). Scoring within national norms has also been found in studies that recruited children with LI and examined reading (Snowling et al., 2000, 2016). Furthermore, nonsignificant findings have been reported. For example, Eisenmajer et al. (2005) found the DD group did not significantly differ from controls on measures of language in post hoc analysis (Eisenmajer et al., 2005; this study also included a DD + LI group that did struggle with language). Catts et al. (2005) also found the LI group did not significantly differ from controls on measures of reading (Catts et al., 2005). Together, these results demonstrate that DD and LI are associated, but that children with DD vary in language ability and may often not be as impaired in language as reading (Adlof & Hogan, 2018).

The varying extent of language difficulties in children with DD was highlighted by McArthur et al. (2000) when they examined the DD-LI comorbidity literature. When they examined studies recruiting children classified as having DD—some children also had comprehension problems—they found 13% to 63% of those children had comorbid language difficulties. When they examined studies recruiting children classified as having LI, they found 12.5% to 85% of children had reading difficulties (McArthur et al., 2000). In addition, McArthur et al. (2000) examined the overlap between DD and LI within their own sample of 212 children, of which 110 children had DD and 102 had an LI. They reported approximately 53% of children with DD could be classified as LI or vice versa (McArthur et al., 2000), which was similar to Catts’s (1993) and Ramus’s (2013) estimates (Catts, 1993; Ramus et al., 2013). These findings differed from those of Catts et al. (2005), however, who reported approximately 30% of their sample met criteria for DD and LI; Aram & Nation (1980) who reported 40% of their sample met criteria for both; and, more recently, Snowling et al. (2016), who reported that 41% of their persistent LI group met criteria for DD.

The variability in overlap can be explained by the heterogeneous nature of language-based disorders, age of assessment, and recruitment methodology, whether it be population-based versus clinical samples or recruitment for language versus reading difficulties (Catts et al., 2005; Ramus et al., 2013; Snowling et al., 2016). Ramus et al. (2013) recruited a clinical sample, which may explain the higher estimates in their sample compared with Catts et al. (2005), who recruited a population-based sample. McArthur et al. (2000) recruited from both reading clinics and schools and also obtained higher estimates.

In summary, studies have examined the relationships between DD-ELD with differing findings, and varying degrees of language difficulties in children with DD have been reported (Pennington & Bishop, 2009; Rescorla, 2011). We sought to examine the association between DD and ELD and contribute to understanding the extent of language difficulties in children with DD, using a large, well-characterized sample of children selected for reading difficulties, and their siblings (*N* = 647).

To examine these relationships, we proposed a total of four aims. The initial aims were to determine (1) whether there is a significant association between DD and ELD and (2) the association between DD and current language difficulties, as well as report the overlap between DD and LI within our sample. Based on the literature, we hypothesized there would be a significant association between DD and ELD and DD and current language difficulties, at age of assessment. Therefore, we proposed Aims 3 and 4 to examine these relationships. These aims were to determine (3) the relationship between ELD and measures of reading and language component skills, and (4) whether early and current language skills predicted decoding skills. Note, Aim 4 acted to bring each of the three descriptive aims together to determine how they contribute to variance in decoding skills.

To address these aims, we used multiple sources of information and statistical analyses. We queried children’s parents on early and current language to obtain a measure of perceived language difficulties. We also directly assessed the children on reading and language skills on quantitative, standardized tests. To compare with previous studies, we grouped participants into categorical reading groups. We also analyzed reading as a continuous measure to allow more variation and increase power. As such Aim 1 was tested using parent report of early language and both (1a) categorical and (1b) continuous reading outcome variables. Aim 2 was tested using parent report and quantitative measures of language as well as both (2a) categorical and (2b) continuous reading outcome variables. Aim 3 was tested by grouping participants based on early language and assessing quantitative tests of reading and language. Finally, Aim 4 was tested using multivariable linear regression to understand how the language variables contribute to decoding skill.

## Method

### Participants

A family-based cohort was recruited from Ontario, predominantly from the Greater Toronto Area as part of a genetic study that has been described elsewhere (Couto et al., 2010; Elbert et al., 2011; Price et al., 2020; Tran et al., 2014). Primary participants—probands—were children, ages 6 to 16 (*M*_age_ = 10) years old, who struggled with reading and were classified as having DD (*n* = 492). Siblings meeting the age criteria, regardless of reading ability, were included as well (*n* = 182). Nonaffected siblings of children with DD share genetic risk for reading difficulties and are thus more likely to have language and literacy problems compared with typically developing children with no family history of DD (Lyytinen et al., 2005; Snowling & Melby-Lervag, 2016). However, the nonaffected siblings share their home and community environments with their sibling with reading difficulties, thereby controlling for differences in socioeconomic status and literacy environment between reading groups (Snowling & Melby-Lervag, 2016).

The children’s parents reported their families’ ethnicities. The families were mainly of European Caucasian descent (87%). The remaining families reported their ethnicities as Asian or Asian and non-Asian mixed (~4%), Indigenous or Indigenous and non-Indigenous mixed (~4%), Caribbean or Caribbean and non-Caribbean mixed (~2%), and other descent (~3%).

### Testing Procedures and Sample Selection

Families interested in participating in the study completed intake interviews. Children were excluded if there was evidence from parent or teacher report of symptoms of psychiatric disorders or medical conditions that would interfere with education. Children were excluded for the following: symptoms of autism spectrum disorder or pervasive developmental delay, bipolar disorder, psychosis, tics, or Tourette syndrome (Couto et al., 2010). Children were also excluded if they had had a head injury, were hearing impaired, or if their birth weight was less than the third percentile for gestational age. Participants were native English speakers or had attended an English-speaking school for ⩾5 years.

In addition to the intake interviews, parents and teachers were further interviewed on or before assessment day. A structured parental interview was used to obtain information on their child’s behavior and development, which included the Parental Childs Interview for Psychiatric Symptoms (PChIPS; Weller et al., 2000). In addition, parents completed standardized questionnaires, including the Ontario Child Health Study Survey Diagnostic Instrument (Offord et al., 1989) and the Conners’ Rating Scale (Conners, 1997). The teachers were interviewed using the Teacher Telephone Interview (TTI; Tannock et al., 2002). The TTI queries the child’s academic performance, attention, behavior, and additional symptoms of psychiatric disease. If parent or teacher interviews identified environmental factors or psychiatric conditions that would interfere with reading development, children were further excluded at that time.

Children meeting entry criteria participated in a full-day assessment, including a test of intelligence and multiple measures of reading and language development. Children were tested by psychometrists with experience in assessing children with learning disabilities. The children were administered the tests in the same order to ensure each child in the study was completing the same test at the same time of day. Many breaks, including a lunch break, were given to each child to avoid fatigue.

Intellectual function was examined. Children were excluded, after assessment, for demonstrating below-average intelligence quotient (IQ) functioning defined as a score of less than 80 on either of the Verbal or Performance domains from the Wechsler Intelligence Scale for Children–Third Edition (WISC-III; Wechsler, 1991) or either Verbal Comprehension or Perceptual Reasoning on the Wechsler Intelligence Scale for Children–Fourth Edition (WISC-IV; Wechsler, 2003). The WISC-III was used to test the first 326 families in the study, and then the updated version, WISC-IV, was used for the remaining families to comply with clinical best practice.

Reading skills were quantitatively evaluated using three standardized reading subtests: Word Identification (ID) and Word Attack (WA) from the Woodcock Reading Mastery Test–Revised (WRMT-R; Woodcock, 1987), and the Reading subtest of the Wide Range Achievement Test–Third Edition (WRAT-3-R; Wilkinson, 1993). The WRMT-R-ID and WRAT-3-R subtests are similar because they assess the child’s ability to read single real words of increasing difficulty. The WRAT-3-R subtest additionally includes recognizing and pronouncing letters. The WRMT-R-WA tests phonetic decoding of nonwords. When evaluating reading, results were compared with standard scores (SS) of chronological-age peers.

The SS of the reading decoding subtests were averaged, and a score was given to each child. Based on this average, we separated our sample into three categorical reading groups to compare with previous studies (Aim 1a and 2a). Participants were classified as having DD if they scored 1.0 standard deviation below the normative value (population mean SS = 100, *SD* = 15) on the average of all three measures (SS ≤ 85; Tran et al., 2014). Although there is no specific cutoff that delineates DD from competent readers, 1 *SD* cutoff criteria is clinically meaningful, often used in research studies, and supported by previous studies investigating the relationship between DD and language (Catts et al., 2005; Colenbrander et al., 2018; McArthur et al., 2000; Snowling et al., 2003). Participants were classified as being at *intermediate readers* (IR) level if their average SS was from 85 to 100, including 100, and as being at *skilled readers* (SR) level if their average SS was greater than 100. We also used the average scores as a continuous variable for additional analyses (Aim 1b and 2b).

Early and current language were queried using the Family and Household questionnaire, with questions developed by the Chedoke-McMaster Child and Family Centre for the Ontario Child Health Study (OCHS; Offord et al., 1989). The OCHS is a parent report questionnaire that surveyed over 2,000 households with children on matters related to early development, general family functioning, emotional and behavioral disorders, schooling, and health (Boyle et al., 1993; Byles et al., 1988; Offord et al., 1989). The questionnaire pertaining to general family functioning was previously tested, correlated, and validated using two other established self-report assessment measures, the Family Unit Inventory and the Family Adaptability and Cohesion Evaluation Scale (FACES II; Olson et al., 1979), with nonclinical individuals. Reliability measurements were Cronbach’s alpha (.86) and split-half correlation (.83; Byles et al., 1988). The questionnaire pertaining to emotional and behavioral disorders was modeled using the *Diagnostic and Statistical Manual of Mental Disorders–Fifth Edition* (*DSM*-5) and also tested for validity and reliability (Boyle et al., 1993, 2018). The questionnaire materials and findings of the OCHS are well established and have been published by *Statistics Canada* (https://www.statcan.gc.ca/eng/survey/household/3824). Questionnaire questions used in this study are presented in Table 1. We will be referring to these as parent report responses.

**Table 1.** Parent Report Form of Language Difficulties by Reading Group.

Question	DD	%	IR	%	SR	%	χ^2^	*p*-value
Early language acquisition 1. At what age was your child able to put at least three words together in a phrase for the first time?^a^								
A) Less than 2 years	173/309	56.0	159/254	62.6	64/87^[Table-fn table-fn2-00222194211006207]^	73.6	χ^2^(6) = 17.5	7.6 × 10^−3^
B) 2–2.5 years	85/309	27.5	66/254	26.0	20/87	23.0		
C) 2.5–3 years	35/309	11.3	25/254	9.8	3/87	3.4		
D) More than 3 years old	16/309^b^	5.2	4/254	1.6	0/87	0		
Current expressive language ability 2. Does your child have difficulties expressing himself/herself, producing sentences or carrying out conversations?^a^								
	78/322	24.2	44/258	17.0	11/92	12.1%	χ^2^(2) = 8.8	.01
Current receptive language ability 3. Does your child understand directions as well as other children her/his age?^c^								
	64/319	20.0	48/257	18.7	11/91	12.0	χ^2^(2) = 3.0	.22

*Note*. DD = developmental dyslexia; IR = intermediate readers; SR = skilled readers; AR = adjusted residual.

a Ratio reflects the parents who responded “yes”/total parent responses for a given group. ^b^ Significantly contributed to the test (AR = 2.95, *p* = .003). ^c^Ratio reflects the parents who responded “no” to this particular question / total.Denominators may differ when parents did not respond to the question.

Current language skills were also directly assessed using two quantitative, standardized subtests: the Clinical Evaluation of Language Fundamentals–Third Edition, Expressive Language subtest (CELF-3-EL) and the Clinical Evaluation of Language Fundamentals–Third Edition, Receptive Language subtest (CELF-3-RL; population mean SS = 100, *SD* = 15; Semel et al., 1995). Like reading, when evaluating current language skills, results were compared with SS of chronological-age peers (Lahey, 1990).

To calculate overlap between DD and LI, we used a cutoff of 1 *SD* below the mean (*M* = 100, *SD* = 15) on the CELF-3 measures to represent impairment. This cutoff criteria is clinically meaningful and supported by previous studies (Kamhi & Catts, 1986; McArthur et al., 2000; Snowling et al., 2016).

Children gave verbal assent or written consent to participate in the study and parents gave written informed consent for their children. Protocols were approved by the Hospital for Sick Children and the University Health Network Research Ethics Boards.

### Statistical Analysis

Statistical analysis was conducted in R version 3.3.0 (https://cran.r-project.org/) with its integrated development environment R Studio (www.rstudio.com). Differences between reading groups on parent report responses, all of which were categorical variables, were analyzed using the chi-square analysis. The quantitative reading and language measures (WRAT-3, WRMT-R-WA, WRMT-R-ID, CELF-3-EL, and CELF-3-RL) were continuous variables. They were plotted to assess normality and were found to be acceptable for skewness and kurtosis. Differences between reading groups on quantitative reading and language measures were analyzed using analysis of variance (ANOVA), or Welch’s ANOVA, when homoscedasticity was not met. A Bonferroni correction was used to adjust for the number of tests performed (threshold for significance at *p* = .05 / 5 = .01, not including reading measures). The same methods were used for the early language groups (threshold for significance *p* < .007; Aim 3).

Post hoc testing for the chi-square test was completed using adjusted residuals (AR), which are representative of *z*-scores (reported in Supplementary Materials; Beasley & Schumacker, 1995). A *p*-value was generated in R from the AR. This does not inherently correct for multiple testing, and therefore a Bonferroni correction was used to avoid Type I errors (Beasley & Schumacker, 1995). Post hoc pairwise comparisons for the ANOVA were completed using Tukey’s HSD test, or Games-Howell when homoscedasticity was not met. R corrects for multiple testing within Tukey’s HSD and the Games-Howell test. The *p*-value thresholds for significance are *p* < .004 and *p* < .02 for Supplemental Table S1, *p* < .05 for Supplemental Table S2, and *p* < .0125 for Supplemental Table S3.

The continuous measure of reading was also examined with the parent response questions and with the quantitative measures of language. The outcome variable, the averaged reading scores, was regressed onto each explanatory variable of interest in a univariate analysis (Aim 1b and 2b).

Next, a multivariable linear regression analysis was performed to determine whether parent report responses regarding early language and the quantitative measures of current language predict reading ability. Variables that reached significance in the univariate analyses were included in this analysis. Collinearity between explanatory variables was tested using correlation tests, by visually assessing scatterplots and by evaluating the variance inflation factor of each variable in the final model (multicollinearity). The explanatory variables put into the model following this approach were (a) the age at which the child put three words together in a phrase for the first time, (b) CELF-3 Expressive Language, and (c) CELF-3 Receptive Language. The parent report of expressive language was not included in the model due to its similarity to CELF-3-EL. The model was assessed by examining the adjusted *R*-squared and the theoretical quantiles plotted against the standardized residuals.

To further examine the relationship between early language, current language, and reading skills, an intervening model using linear regressions was performed. The intervening model involved three steps. Step 1 was to determine whether there is a significant association between reading and early language skills. Step 2 was to determine whether there is a significant association between early and current language. Finally, if Steps 1 and 2 were significant, Step 3 was to determine whether language skills diminished the effect of early language acquisition on reading.

## Results

The reading groups consisted of 323 children classified as having DD, 259 children classified as being at the IR level, and 92 children classified as being at the SR level (*N* = 674). A higher ratio of males to females (1.6:1) was seen across all groups (χ^2^ = 0.77, *df* = 2, *p* = .68). All reading groups performed significantly different on reading measures, as expected given that groupings were based on these measures (all three measures *p* < 1.0 × 10^−10^). Post hoc analysis revealed that the DD group had lower scores than the IR and SR groups, and the IR group performed significantly lower than the SR group (see Table 2).

**Table 2.** Quantitative Measures of Reading and Language by Reading Group.

Quantitative measure	DD (*n* = 323)	IR (*n* = 259)	SR (*n* = 92)	*F*-value	*p*-value
WRAT-3-R	77.0 ± 8.5	93.7 ± 6.6	109.5 ± 7.3	*F*(2, 257.6) = 737.6	<1.0 × 10^−10^
WRMT-R-WA	74.9 ± 9.8	90.5 ± 6.3	105.7 ± 7.9	*F*(2, 249.3) = 532.8	<1.0 × 10^−10^
WRMT-R-ID	73.8 ± 10.5	92.2 ± 6.2	108.3 ± 8.2	*F*(2, 246.9) = 616.9	<1.0 × 10^−10^
CELF-3-EL	85.9 ± 13.2	95.3 ± 12.8	102.7 ± 12.8	*F*(2, 662) = 73.8	<1.0 × 10^−10^
CELF-3-RL	88.9 ± 13.8	96.1 ± 13.7	105.4 ± 13.9	*F*(2, 664) = 55.1	<1.0 × 10^−10^

*Note.* Standard score mean and standard deviation given for each reading group. All groups contributed to significance in test. DD = developmental dyslexia; IR = intermediate readers; SR = skilled readers; WRAT-3-R = Reading subtest of the Wide Range Achievement Test–Third Edition; WRMT-R = Woodcock Reading Mastery Test–Revised; WA = word attack; ID = word identification; CELF-3= Clinical Evaluation of Language Fundamentals–Third Edition; EL = Expressive Language subtest. RL = Receptive Language subtest.

The first aim of this study is to address whether there was an association between DD and ELD. For the parent report question addressing early language acquisition, stated as “At what age was your child able to put at least three words together in a phrase for the first time?” we found a statistically significant difference among reading groups (χ^2^ = 17.5, *df* = 6, *p* = 7.6 × 10^−3^; see Table 1), indicating there is an association between decoding skills and the age children put their first words together. The post hoc analysis revealed that children with DD put three words together at a later age than children with less reading difficulty (see Table 1; Supplemental Table S1), thereby demonstrating an association between DD and ELD (Aim 1a). The univariate analysis also identified a significant association, with children with low average reading scores putting words together at later ages (*p* value = 2.64 × 10^−5^, *r*^2^ = .027, see Table 3; Aim 1b).

**Table 3.** Univariate Analysis With Continuous Measure of Reading.

Outcome variable	Explanatory variable	β	*SE*	*t*	Adjusted *R*^2^	*p*-value
Decoding^a^	Age put words together	2.79	.66	4.23	.027	2.64 × 10^−5^
Decoding	Answered “No” to expressive difficulties	5.21	1.32	3.94	.02	8.89 × 10^−5^
Decoding	Answered “No” to receptive difficulties	2.15	1.35	1.60	.004	.11
Decoding	CELF-3-EL	0.47	.03	14.42	.25	<1.0 × 10^−10^
Decoding	CELF-3-RL	0.41	.03	12.64	.20	<1.0 × 10^−10^

*Note.* CELF-3 = Clinical Evaluation of Language Fundamentals–Third Edition; EL = Expressive Language subtest. RL = Receptive Language subtest.

a Reading (decoding) score is based on average of the three reading measures.

The second aim of the study was to test the association between DD and current language difficulties and report the overlap. Beginning with the parent report questions, for the question addressing expressive language, stated as “Does your child have difficulties expressing himself/herself, producing sentences or carrying out conversations?” we found a significant difference among reading groups, just meeting the threshold for significance (χ^2^ = 8.8, *df* = 2, *p* = .01; see Table 1). This indicated there is an association between decoding skills and expressive language skills. The post hoc analysis revealed that children with DD reported more expressive language difficulties (see Table 1; Supplemental Table S1), thereby demonstrating an association between DD and ELD (Aim 2a). We found 24% of parents of children with DD reported expressive language difficulties. For the question aimed at assessing receptive language, stated as “Does your child understand directions as well as other children his or her age?” we found no evidence of association between decoding and receptive language skills (χ^2^ = 3.0, *df* = 2, *p* = .22; see Table 1; Aim 2a). However, we found that 20% of parents reported receptive difficulties in their child in the DD group.

When we performed univariate analysis with the continuous reading scores and the parent report of expressive language, we found a significant association (*p*-value = 8.89 × 10^−5^, *r*^2^ = .02, see Table 3; Aim 2b). This was not the case for the univariate analysis with the parent report of receptive language, where no association was found (*p* = .11, *r*^2^ = .004, see Table 3; Aim 2b).

Next, using current quantitative measures of language, we identified a significant difference among reading groups for both CELF-3 measures (*p* < 1.0 × 10^−10^ for both; see Table 2). Post hoc analysis revealed that the DD group performed significantly lower than the IR and SR groups, and the IR group performed significantly lower than the SR group (see Table 2), thereby demonstrating the association between DD and language difficulties (Aim 2a). Then, using a cutoff of 1 *SD* below the mean on language measures as an indicator of impairment, we found the overlap between DD and quantitative expressive language difficulties to be 46% and DD and quantitative receptive language difficulties to be 36%. The univariate analysis analyzing the continuous reading scores and quantitative language also identified a significant association (*p* < 1.0 × 10^−10^ for both, *r*^2^ = .25 and .20 for the CELF-3-EL and CELF-3-RL respectively, see Table 3; Aim 2b).

We hypothesized there would be an association between (a) DD and ELD and (b) DD and current language difficulties and therefore proposed the third and fourth aims to examine these relationships. Indeed, we found an association between these difficulties. As such, in Aim 3, we sought to examine the relationship between the different reading and language difficulties and ELD, to tease apart whether it was one aspect of reading driving the association and to test the relationship between ELD and current language difficulties. It should be noted early language was a parent response question and we did not have early childhood language measures to support parental report.

In the third aim to determine whether there is an association between ELD and reading and language difficulties, we created groups based on the parental responses to the early language question. The groups consisted of 396 children who were less than 2 years old when they first put three words together, 171 who were 2 to 2.5 years old, 63 who were 2.5 and 3 years old, and 20 who were more than 3 years old. The ratio of males to females was higher in the later-to-speak language groups (χ^2^ = 13.25, *df* = 3, *p* = .004).

For the analysis of ELD and the reading measures, we found a significant difference among early language groups for WRAT-3-R (*p* = 8.77 × 10^−6^) and WRMT-R-WA (*p* = 3.0 × 10^−4^, see Table 4). Post hoc analysis revealed that children who put three words together at a later age scored lower on the WRAT-3-R or WRMT-R-WA subtest compared with children who put three words together at an earlier age (see Supplemental Table S2). For WRMT-R-ID, the ANOVA fell short of significance (*p* = .01; see Table 4).

**Table 4.** Quantitative Measures of Reading and Language by ELD Group.

Three words together in a phrase						
Quantitative measure	<2 years (*n* = 396)	2–2.5 years (*n* = 171)	2.5–3 years (*n* = 63)	>3 years (*n* = 20)	*F*-value	*p-*value
WRAT-3-R	89.4 ± 14.2	87.3 ± 13.4	83.4 ± 10.1	77.1 ± 12.4	*F*(3,78.3) = 10.3	8.77 × 10^−6^
WRMT-R-WA	86.1 ± 14.1	84.7 ± 13.2	82.0 ± 12.1	73.9 ± 14.5	*F* (3, 643) = 6.3	3.0 × 10^−4^
WRMT-R-ID	86.6 ± 15.6	85.3 ± 15.3	82.3 ± 12.6	77.2 ± 13.3	*F* (3, 643) = 3.6	.01
CELF-3-EL	93.9 ± 13.7	90.1 ± 14.0	87.5 ± 14.9	80.1 ± 14.6	*F* (3, 638) = 10.5	9.86 × 10^−7^
CELF-3-RL	95.5 ± 14.1	92.3 ± 15.4	91.8 ± 14.6	84.5 ± 16.4	*F* (3, 640) = 5.4	1.12 × 10^−3^

*Note.* Standard score mean and standard deviation given for each ELD group. See Supplemental Table S2 for contributions to significance in test. ELD = early language delay; WRAT-3-R = Reading subtest of the Wide Range Achievement Test–Third Edition; WRMT-R = Woodcock Reading Mastery Test–Revised; WA = word attack; ID = word identification; CELF-3 = Clinical Evaluation of Language Fundamentals–Third Edition; EL = Expressive Language subtest. RL = Receptive Language subtest.

For the parent report question addressing current expressive language, we found a significant difference among early language groups (χ^2^ = 47.4, *df* = 3, *p* = 2.92 × 10^−10^; see Table 5). Post hoc analysis revealed that all early language groups, except 2 to 2.5 years, significantly contributed to the test (see Supplemental Table S3). For the parent report question addressing current receptive language, no significant association was found (χ^2^ = 10.1, *df* = 3, *p* = .02; see Table 5).

**Table 5.** Parent Report Form of Language Difficulties by ELD Group.

Three words together in a phrase						
Question	<2 years	2–2.5 years	2.5–3 years	>3 years	χ^2^	*p*-value
3. Difficulties expressing himself/herself, producing sentences or carrying out conversations^a^	51/395^b^ (12.9%)	40/171 (23.4%)	23/63^b^ (36.5%)	12/19^b^ (63.2%)	χ^2^(3) = 47.4	2.92 × 10^−10^
4. Understands directions as well as other children her/his age^c^	68/392 (17.3%)	28/170 (16.5%)	17/62 (27.4%)	8/20 (40.0%)	χ^2^(3) = 10.1	.02

*Note.* ELD = early language delay; AR = adjusted residual.

a Ratio reflects the parents who responded “yes”/total parent responses for a given ELD group. ^b^ Significantly contributed to the test (AR = −5.25, 3.60, 4.89, all *p* < .025, respectively). ^c^ Ratio reflects the parents who responded “no”/total.

For both the current quantitative language measures, CELF-3-EL and CELF-3-RL, we found a significant difference among early language groups (*p* = 9.86 × 10^−7^, *p* = 1.12 ×10^−10^, respectively, see Table 4). Post hoc testing using Tukey’s HSD revealed that children who put three words together at a later age had lower scores on the expressive and receptive language measure than children who put three words together at an earlier age (see Supplemental Table S2).

The fourth aim of the study was to determine whether early and current language skills predict reading skills. The CELF-3-EL and CELF-3-RL measures were correlated with the reading measures with *r* = .49, .44 (*p* < 1 × 10^−10^). ELD was correlated with reading measures with *r* = .16 (*p* = 2.64 × 10^−5^). When the explanatory variable querying early language and the CELF-3 were both put into the linear regression, the regression model was significant, *F*(3, 635) = 78.22, *p* < 1.0 × 10^−10^, with an *R*-squared of .27 (see Table 6). The CELF-3 variables significantly contributed to reading, with each incremental increase in CELF-3-EL or -RL causing the average reading score to increase. The early language variable was no longer significant by multivariable linear regression.

**Table 6.** Multivariable Linear Regression of Reading Ability Based on Early and Current Language Difficulties.

Variable	β	*SE*	*t*	*p*-value	95% confidence interval
Intercept	36.14	3.47	10.43	<1.0 × 10^−10^	[29.33%, 42.94%]
Age put words together (1–4)	.99	.58	1.70	.09	[–.16%, 2.14%]
CELF-3-EL	.32	.05	7.15	<1.0 × 10^−10^	[.23%, .41%]
CELF-3-RL	.18	.04	4.21	2.93 × 10^−5^	[.09%, .27%]

*Note.* CELF-3 = *Clinical Evaluation of Language Fundamentals–Third Edition; EL = Expressive Language subtest. RL = Receptive Language subtest.*

As stated previously, ELD was significantly associated with reading difficulties (Aim 1a and 1b). Early language groups were also shown to be significantly associated with the individual WRAT-3-R and WRMT-R-WA reading subtests, parent report of expressive language, and both CELF-3 quantitative language measures (Aim 3). As such, we sought to test whether early language was not significant in multivariable linear regression because the CELF-3-EL or CELF-3-RL variables acted as intervening variables. Said in another way, we sought to see whether the early language variable did not explain any unique variation in reading when the CELF-3-EL/RL variables were present.

**Figure 1.**
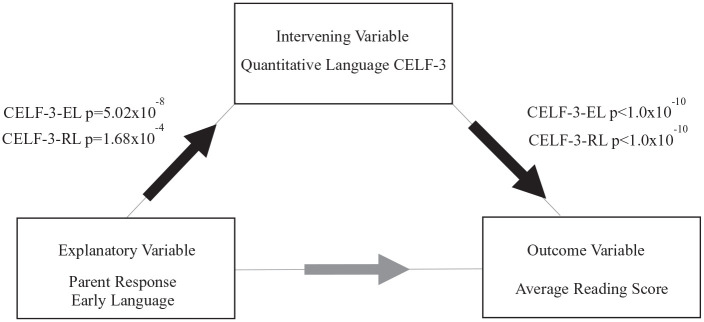
Intervening model for language and reading. *Note.* Proposed model for the relationship between early language, current language, and reading. Early language is measured by parent report and current language and reading by quantitative, standardized tests. Early language acquisition predicted current language skills, which predicted reading skills. Early language is also associated with reading, but this effect is diminished with current language included in the model. CELF-3 = Clinical Evaluation of Language Fundamentals–Third Edition; EL = Expressive Language subtest. RL = Receptive Language subtest.

The intervening model involved three steps. First, based on the univariate analysis, we determined early language significantly predicted reading (*p* = 2.64 × 10^−5^, *r*^2^ = .027, see Table 3). Next, by univariate analysis, we determined early language significantly predicted CELF-3-EL and CELF-3-RL (*p* = 5.02 × 10^−8^, 1.68 × 10^−4^, *r*^2^ = .04, .02, respectively, see Supplemental Table S4). Finally, we assessed whether the language measures intervene with early language and diminish its effect on reading in linear regression. Indeed, the early language *p*-value became less significant with either CELF-3-EL or CELF-3-RL included in the model (ELD *p* = 9.50 × 10^−2^, 5.34 × 10^−3^, *r*^2^ = .25, .21, respectively, see Supplemental Table S4). Therefore, the results indicated that the explanatory variable querying ELD was not a significant predictor of reading skills in the model because its relationship with reading was explained through the CELF-3-EL variable and marginally through the CELF-3-RL (see Supplemental Table S4). In conclusion, we determined early language significantly predicted current language skills, which significantly predicted reading skills (see Figure 1).

## Discussion

This study examined the association between DD and language difficulties in school-age children recruited for reading difficulties and their siblings. Consistent with our hypothesis, we found a significant association between DD and ELD and DD and language difficulties. These results are supported by those of Preston and colleagues (2010), who also reported a significant association between word reading in school-age children and ELD, as well as the body of research associating DD and language difficulties (Bishop & Adams, 1990; Catts et al., 2002, 2005; Goulandris et al., 2000; McArthur et al., 2000; Snowling et al., 2000; Stark et al., 1984; Tomblin et al., 1997). We also determined that ELD significantly predicted current language ability, which predicted reading skills. Below, we discuss the strengths and limitations of this study, in addition to an explanation of each of our findings in light of these factors.

The first strength of this study is the recruitment of a large clinical sample of school-age children with reading difficulties and their siblings. We used specific, strict exclusion criteria to recruit a sample of children with reading difficulties, with no comorbid neurodevelopmental or intellectual disabilities. The recruitment of a sample this size is uncommon in reading disabilities and is an asset to understanding DD. The children in our study were recruited based on current reading problems and were more likely to have had language-based problems, whereas children in longitudinal studies may resolve their language-based deficits by the time they reach school age and score within the normal reading range. As such, we were able to examine language relationships in the context of moderately/severely affected children. Second, children were tested by trained psychometrists over a full-day assessment on multiple measures of language and reading, using tests with established validity and reliability. For current language ability, we had quantitative measures of expressive and receptive language in addition to parent report. For reading, we had three measures of decoding.

The first limitation of this study is categorizing the continuous reading scores. This is a limitation because it decreases “important information pertaining to both severity within the disorder and variability in subthreshold symptomatology” (Willcutt et al., 2010, p. 4). Furthermore, the degree of overlap when categorizing variables is influenced by the correlation between variables (Branum-Martin et al., 2013). Be that as it may, categorizing learning disability measures is an established method, which we adopted to be comparable with previous studies (Catts et al., 2005; Duff et al., 2015; Goulandris et al., 2000; Lewis et al., 2015; Lyytinen et al., 2005; Preston et al., 2010). It allowed us to use descriptive statistics, ANOVA, or chi-square and thus to delineate characteristics specific to a group, which would not have been possible with linear regression. Furthermore, it is critical for clinical diagnosis and the treatment of children. Because of the expense of categorizing a continuous variable, we also included the continuous measure of reading in the univariate analyses and the multivariable linear regression to maintain the variation and improve power (Aim 4).

The second limitation of this study is the use of parent report for early language. Repeated quantitative measures of language over a range of ages would have enabled us to better understand the developmental trajectory of children at risk of DD. That being said, in the study of language-based difficulties, parents are considered a common and important resource due to the dynamicity of early language acquisition (Diamond & Squires, 1993). Parents observe their child in a variety of environments and offer an extensive and unique knowledge as to their child’s capabilities in a stress-free context. They help researchers understand the child’s language history, as it is expensive and sometimes unfeasible to do formal testing at multiple ages. Furthermore, studies have demonstrated strong correlations between professional and parent-based reports (Boudreau, 2005; Dinnebeil & Rule, 1994; Rescorla, 2011).

Returning to the findings, in the first aim, we report a significant association between DD and ELD. Despite the consensus that ELD is a risk factor for reading difficulties (Rescorla, 2011), multiple studies have reported no significant association between DD and ELD (Dale et al., 2014; Duff et al., 2015; Paul et al., 1997; Poll & Miller, 2013; Rescorla, 2005, 2009). The significant association we report may be because of our sample’s DD clinical ascertainment, age at assessment, or sample size. Studies reporting no significant association largely sampled younger children with ELD, or history of ELD, and followed them longitudinally. As such, early language difficulties may have recovered by reading age (Dale et al., 2014). Furthermore, the size of our study may have increased power to identify relationships that were not apparent in smaller samples. We were therefore able to validate weaker associations for ELD and reading (Rescorla, 2011).

In the second aim, we found an association between DD and language difficulties. We found a significant association between categorical and continuous reading and quantitative measures of language, with means for each of the three groups still in the normative range (e.g., DD group CELF-3-EL SS = 85.9, CELF-3-RL = 88.9), albeit on the lower edge. Finding DD group language scores within the normative range is similar to previous reports (Alt et al., 2017; Catts et al., 2005), although sample recruitment and individual reading and language tests play a role. We also found a significant association between DD and the parent report for expressive language, but not for receptive language. The CELF-3-EL and the expressive language question, and, the CELF-3-RL and receptive language question had the following respective significant correlations: .27 (*p* = 4.48 × 10^−12^) and .18 (*p* = 2.45 × 10^−6^; significance threshold = 0.05 / 2 = 0.025). Parents may have not been able to recognize receptive language difficulties because they were not discernible from peers.

Regarding the overlap between DD and language difficulties, we found parent endorsement for language for the DD reading group was on the lower end of what has previously been reported. We report 24% overlap for DD and expressive difficulties and 20% for DD and receptive difficulties in our sample. However, when we measured language quantitatively using the CELF-3, we found DD and LI overlap to be higher at 36% to 46%. We demonstrated within our own study how the definition of language, cutoffs, and groupings can influence the comorbidity outcomes (Adlof & Hogan, 2018). The fact we had a clinical sample for DD and not a clinical sample for LI or a population sample affected these estimates.

Next, in Aim 3, we examined reading and language in children with reported ELD. We found a significant association between ELD and the WRAT-3-R and WRMT-R-WA subtests, but the WRMT-R-ID was not significant after accounting for the multiple tests. The WRMT-R-ID is correlated with WRAT-3-R (Pearson’s *r* = .79) as they both examine single-word reading with increasingly difficult items. The WRAT-3-R additionally includes recognition and pronunciation of letters. It is unclear whether the different findings reflect the stringent Bonferroni correction with results from the WRMT-R-ID failing to meet significance or attributes of the tests, including the differing word selection with different difficulties.

We found association between ELD and language difficulties. Both CELF-3 scores were significantly associated with ELD, with the average for each of the groups lower for the CELF-3-EL than for the CELF-3-RL. The parent report supported a significant relationship between expressive language difficulties, with a higher prevalence of expressive language difficulties in the children with ELD. This finding suggested there is a subset of children in our DD sample who first put three words together at a later age, had lower expressive language scores, and more reading difficulties. Taken together with results from the first and second aims, this led us to the multivariable linear regression to test if early and current language abilities could predict reading skills.

Multivariable linear regression demonstrated that language is a statistically significant predictor of reading score. The CELF-3-EL and CELF-3-RL were the significant predictors in the regression analysis. Early language was significant in the univariate analysis but not in the multivariable regression analysis when CELF-3-EL and CELF-3-RL were included. Further analyses indicated that explanatory variable querying early language did not explain any unique variation in the reading outcome when the CELF-3-EL or CELF-3-RL variable was present. We found ELD predicts language skills that predict reading skills.

It is known single-word reading allows children to increase their vocabulary knowledge and language abilities (Wise et al., 2007), thereby improving reading fluency and eventual comprehension (Lyon, 2003). We found an increased age at which a child put words together in the DD group, indicating that there were preexisting language difficulties (ELD) prior to reading acquisition. This suggests these children already had deficits before reading, which hindered their later language and reading ability, affecting single-word reading, fluency, and even later comprehension skills.

In summary, our study demonstrated that relative to skilled readers, school-age children with reading difficulties are more likely to have had ELD and current expressive/receptive language problems. These receptive language difficulties may not be apparent to parents because they appear to fall within national norms and potentially are not discernible from peers. Expressive language difficulties were also within national norms but on the lower end. In addition, we found children with ELD are more likely to experience language difficulties, which interfer with their reading. The information found can encourage teachers, service educators, speech/language pathologists, and psychologists to identify more subtle language difficulties in children with DD and to act early to facilitate timely intervention and better outcomes.

## Supplemental Material

sj-docx-1-ldx-10.1177_00222194211006207 – Supplemental material for Language Difficulties in School-Age Children With Developmental Dyslexia Supplemental material, sj-docx-1-ldx-10.1177_00222194211006207 for Language Difficulties in School-Age Children With Developmental Dyslexia by Kaitlyn M. Price, Karen G. Wigg, Virginia L. Misener, Antoine Clarke, Natalie Yeung, Kirsten Blokland, Margaret Wilkinson, Elizabeth N. Kerr, Sharon L. Guger, Maureen W. Lovett and Cathy L. Barr in Journal of Learning Disabilities
