# Supplementary material for: Language Difficulties in School-Age Children With Developmental Dyslexia
Source: J Learn Disabil. 2021 Apr 23;55(3):200–12. doi: 10.1177/00222194211006207 (PMC8996296; doi:10.1177/00222194211006207)
Supplement: sj-docx-1-ldx-10.1177_00222194211006207 – Supplemental material for Language Difficulties in School-Age Children With Developmental Dyslexia [file sj-docx-1-ldx-10.1177_00222194211006207.docx]

JOURNAL OF LEARNING DISABILITIES SUPPLEMENTAL FILE
Table S1. Post Hoc analysis of parent report of early language by reading group
ARTICLE TITLE: Language Difficulties in School-Aged Children with Developmental Dyslexia

| Question |  | DD | IR | SR |
| --- | --- | --- | --- | --- |
| 1. Age child put at least 3 words together |  |  |  |  |
| a) Less than 2 yrs. | Adjusted Residuals P-value | -2.46 0.014 | 0.70 0.483 | 2.60 0.009 |
| b) 2-2.5 yrs. | Adjusted Residuals P-value | 0.66 0.508 | -0.15 0.881 | -0.76 0.450 |
| c) 2.5-3 yrs. | Adjusted Residuals P-value | 1.34 0.180 | 0.10 0.918 | -2.12 0.03 |
| d) More than 3 yrs. | Adjusted Residuals P-value | 2.95 0.003 | -1.78 0.076 | -1.79 0.074 |
| 2. Expressive language | Adjusted Residuals | 2.77 | -1.41 | -2.03 |
|  | P-value | 0.02 | 0.37 | 0.13 |

JOURNAL OF LEARNING DISABILITIES SUPPLEMENTAL FILE
Table S2. Post Hoc analysis of quantitative measures of reading and current expressive language by early language group
ARTICLE TITLE: Language Difficulties in School-Aged Children with Developmental Dyslexia

| Quantitative Measure | Pairwise Comparison | Post Hoc | P-value | Significant |
| --- | --- | --- | --- | --- |
| WRAT-3 | <2 years and 2-2.5 years | Games-Howell | 1.00 | No |
|  | <2 years and 2.5-3 years | Games-Howell | 0.0031 | Yes |
|  | <2 years and >3 years | Games-Howell | 0.0092 | Yes |
|  | 2-2.5 years and 2.5-3 years | Games-Howell | 0.5789 | No |
|  | 2-2.5 years and >3 years | Games-Howell | 0.0613 | No |
|  | 2.5-3 years and >3 years | Games-Howell | 1.00 | No |
| WRMT-R-WA | <2 years and 2-2.5 years | Tukey HSD | 0.640 | No |
|  | <2 years and 2.5-3 years | Tukey HSD | 0.130 | No |
|  | <2 years and >3 years | Tukey HSD | 0.0006 | Yes |
|  | 2-2.5 years and 2.5-3 years | Tukey HSD | 0.570 | No |
|  | 2-2.5 years and >3 years | Tukey HSD | 0.005 | Yes |
|  | 2.5-3 years and >3 years | Tukey HSD | 0.090 | No |
| CELF-3-EL | <2 years and 2-2.5 years | Tukey HSD | 0.020 | Yes |
|  | <2 years and 2.5-3 years | Tukey HSD | 0.004 | Yes |
|  | <2 years and >3 years | Tukey HSD | 0.0001 | Yes |
|  | 2-2.5 years and 2.5-3 years | Tukey HSD | 0.570 | No |
|  | 2-2.5 years and >3 years | Tukey HSD | 0.013 | Yes |
|  | 2.5-3 years and >3 years | Tukey HSD | 0.170 | No |
| CELF-3-RL | <2 years and 2-2.5 years | Tukey HSD | 0.083 | No |
|  | <2 years and 2.5-3 years | Tukey HSD | 0.260 | No |
|  | <2 years and >3 years | Tukey HSD | 0.006 | Yes |
|  | 2-2.5 years and 2.5-3 years | Tukey HSD | 0.100 | No |
|  | 2-2.5 years and >3 years | Tukey HSD | 0.110 | No |
|  | 2.5-3 years and >3 years | Tukey HSD | 0.200 | No |

JOURNAL OF LEARNING DISABILITIES SUPPLEMENTAL FILE
Table S3. Post Hoc analysis of parental report of current language difficulties by early language group
ARTICLE TITLE: Language Difficulties in School-Aged Children with Developmental Dyslexia

| 3 words together in a phrase | | | | | |
| --- | --- | --- | --- | --- | --- |
| Question |  | <2 yrs | 2-2.5 yrs | 2.5-3 yrs | >3 yrs |
| 3. Difficulties expressing  him/herself, producing sentences or carrying out conversations | Adjusted Residuals P-value | -5.25 <0.0001 | 1.52 0.128 | 3.60 0.0003 | 4.89 <0.0001 |

JOURNAL OF LEARNING DISABILITIES SUPPLEMENTAL FILE
Tables S4. Intervening model of early language, current language and reading skills
ARTICLE TITLE: Language Difficulties in School-Aged Children with Developmental Dyslexia

**Step 2:**
Is early language a significant predictor of current language? Yes

| Intervening Variable | Independent Variable | β | SE | t | Adjusted R^2^ | P-value |
| --- | --- | --- | --- | --- | --- | --- |
| CELF-3-EL | Age put words together | 3.82 | 0.69 | 5.52 | 0.04 | 5.02x10^-8^ |
| CELF-3-RL | Age put words together | 2.74 | 0.73 | 3.79 | 0.02 | 1.68x10^-4^ |

**Step 3:**
Are the language measures intervening with the early language measure and therefore diminishing its effect? Yes

| Dependent | Independent Variable | β | SE | t | Adjusted R^2^ | P-value |
| --- | --- | --- | --- | --- | --- | --- |
| Decoding | Age put words together | 0.99 | 0.59 | 1.67 | 0.25 | 9.50x10^-2^ |
|  | CELF-3-EL | 0.45 | 0.03 | 13.75 |  | <1.0x10^-10^ |
| Decoding | Age put words together | 1.68 | 0.60 | 2.80 | 0.21 | 5.34x10-3 |
|  | CELF-3-RL | 0.39 | 0.03 | 12.15 |  | <1.0x10^-10^ |
